# Misdiagnosis of Supraventricular Tachycardia as Panic Attacks: A Case Report Highlighting the Importance of Accurate Cardiovascular Evaluation

**DOI:** 10.7759/cureus.98658

**Published:** 2025-12-07

**Authors:** Sammy Matta, George Mikhail, Daniel Ugona, Roxana Lazarescu, Annie Taffaro

**Affiliations:** 1 Internal Medicine, Wyckoff Heights Medical Center, Brooklyn, USA; 2 Internal Medicine, St. George's University School of Medicine, St. George's, GRD; 3 Internal Medicine, Touro College of Osteopathic Medicine, New York City, USA

**Keywords:** anxiety disorders, arrhythmia diagnosis, atrioventricular nodal reentrant tachycardia, cardiac arrhythmias, cardiac rhythm management, electrocardiogram, misdiagnosis, palpitations, panic attacks, supraventricular tachycardia

## Abstract

Palpitations are a common symptom with diverse etiologies, ranging from benign to life-threatening conditions. One of the challenges in clinical practice is differentiating between cardiac arrhythmias and psychiatric disorders, such as panic attacks, as their symptoms often overlap. This case report presents a 45-year-old female who presented with a history of unexplained panic attacks. She was initially diagnosed with panic disorder. However, she was refractory to all anti-anxiolytic trials. The patient had multiple emergency room (ER) visits for similar episodes, during which all cardiac workups, including electrocardiogram (ECG) and echocardiograms (echo) at a different facility, were negative according to the patient. During an ER visit for a similar episode at Wyckoff Heights Medical Center, she was found to have supraventricular tachycardia (SVT), specifically atrioventricular nodal reentrant tachycardia (AVNRT). The most prominent evidence in her presentation was a sustained heart rate of 217 beats per minute, which ultimately led to performing an ECG. The findings of the ECG were consistent with a diagnosis of narrow complex tachycardia suggestive of SVT. The patient was successfully treated with intravenous diltiazem, and her symptoms resolved promptly. This report underscores the importance of considering cardiac arrhythmias in patients presenting with palpitations, anxiety, and chest discomfort, which may mimic psychiatric conditions even in the absence of abnormalities on initial cardiac workup at presentation. Timely and accurate diagnosis is crucial to avoid delays in appropriate treatment and prevent unnecessary psychiatric interventions. The case highlights the value of thorough cardiovascular evaluation, particularly in patients with recurrent or unexplained episodes of palpitations, as well as the role of advanced diagnostic techniques in improving diagnostic accuracy.

## Introduction

Palpitations are a common symptom that can be associated with a variety of conditions ranging from benign to life-threatening. They can also be caused due to stimulants and some medications [1]. They are defined as “noticeable pounding, fluttering or irregular beating of the heart” [2]. Misdiagnosis of cardiac arrhythmias as psychiatric disorders, such as panic attacks, can delay appropriate treatment. One study from 2019 found that only one out of 10 patients with palpitations who presented to the emergency department (ED) with palpitations were found to have an underlying heart arrhythmia. [2] This demonstrates the rarity of this case while highlighting a possible differential that should be considered for panic attacks that are refractory to anxiolytics. A different study found that 67% of patients are diagnosed as having some type of psychiatric ailment, while in fact they have a primary heart arrhythmia [3]. This directly ties into our patients’ profiles/presentations as they were also misdiagnosed with a psychiatric illness while having an underlying cardiac arrhythmia, which highlights how common this issue is. To further complicate the matter, a 2016 study found that, in patients reporting undocumented tachycardia suspected to be supraventricular tachycardia (SVT), the presence of tachycardia along with chest pain and/or syncope, particularly in those under 40 years old, typically correlated with a negative electrophysiological study (EPS) and did not warrant immediate invasive investigations [4]. All these factors illustrate the difficulties of accurate diagnosis. This is why misdiagnosis can lead to multiple ED visits and adversely affect patient outcomes. However, it is important to point out the difficulty of diagnosing a heart arrhythmia from one isolated ED visit. This is due to the fact that most patients’ arrhythmias have recovered by the time they arrive at the hospital. It is crucial to conduct the required tests to confirm or exclude the diagnosis, despite the challenges involved, as significant adverse events related to SVT are linked to a six-fold increase in long-term mortality risk [5]. These can be tests as simple as a bedside echo or a 24-hour Holter monitor. The misdiagnosis not only delays appropriate treatment but also highlights the potential for serious complications if underlying cardiac issues are overlooked. This case report emphasizes a specific instance of SVT that was initially misidentified as recurrent panic attacks, underscoring the critical need for thorough cardiovascular evaluation in patients presenting with symptoms such as palpitations, anxiety, and chest discomfort, which can often be mistaken for anxiety disorders.

## Case presentation

Case presentation

**Figure 1:**
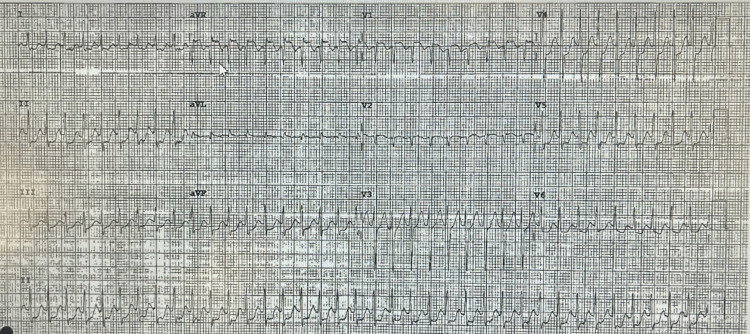
EKG shows regular and narrow complex tachycardia with short RP (most likely atrioventricular nodal reentrant tachycardia (AVNRT)).

**Figure 2:**
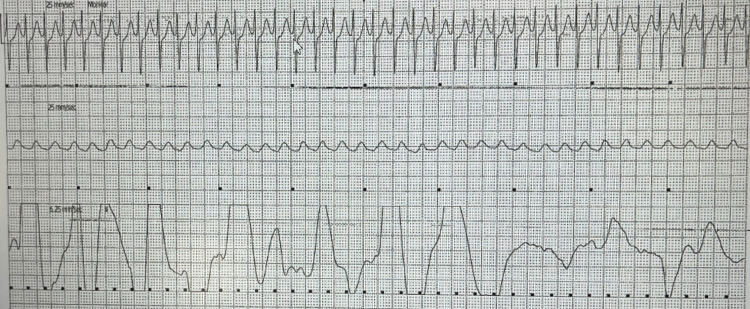
EKG strep shows regular and narrow complex tachycardia with a heart rate of 170 beats per minute.

A 45-year-old female presented to the ED with a past medical history of panic attacks. The patient's chief complaint was recurrent episodes of palpitations, dizziness, and occasional shortness of breath for the past five years. These episodes regularly lasted anywhere from a few minutes to one hour. These episodes, in many instances, were accompanied by a sense of impending doom. This led to a diagnosis of panic attacks by her primary care physician without a formal psychiatric assessment. She was prescribed anxiolytics, which were ineffective in stopping further episodes from occurring. During the ED visit, the patient reported currently having one of these episodes; however, unlike her previous episodes, which were transient and terminated quickly, this episode lasted for five hours. In triage, her heart rate was noted to be 217 beats per minute (BPM), with a regular rhythm. An electrocardiogram (ECG) was performed and revealed narrow complex tachycardia at a rate of 225, consistent with SVT (Figure 1). These differences in rate were due to the examination being done at different times during the patient's stay. The patient was subsequently placed on a telemonitor. An ECG recording from the telemonitor recorded a narrow complex tachycardia (Figure 2).

Diagnosis

The diagnosis of SVT, most likely atrioventricular nodal reentry tachycardia (AVNRT), was confirmed based on the ECG findings and the patient’s clinical history. The initial misdiagnosis of panic attacks was attributed to the overlap in symptoms, such as palpitations and anxiety.

Management and outcome

**Figure 3:**
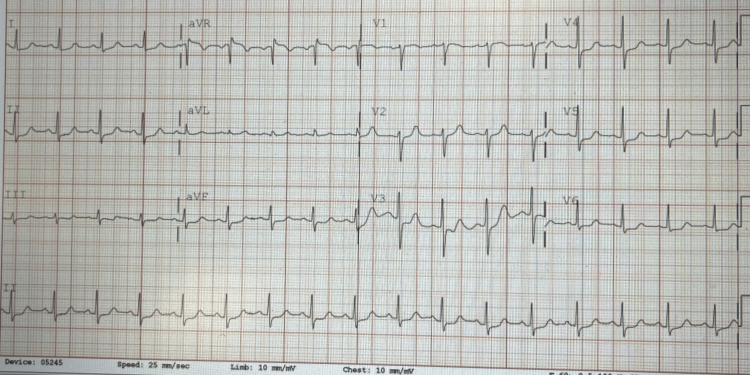
EKG taken after resolution of the symptoms shows normal sinus rhythm. No delta wave can be detected on the EKG.

At the time of presentation, the patient was hemodynamically stable. Vagal maneuvers were performed and failed to terminate the arrhythmia. Diltiazem was chosen as the next step in management. The patient received an intravenous 20 mg bolus of diltiazem. Within minutes, the telemonitor showed SVT termination, and her heart rate decreased to 100 BPM, and the ECG showed normal sinus rhythm (Figure 3).

**Figure 4:**
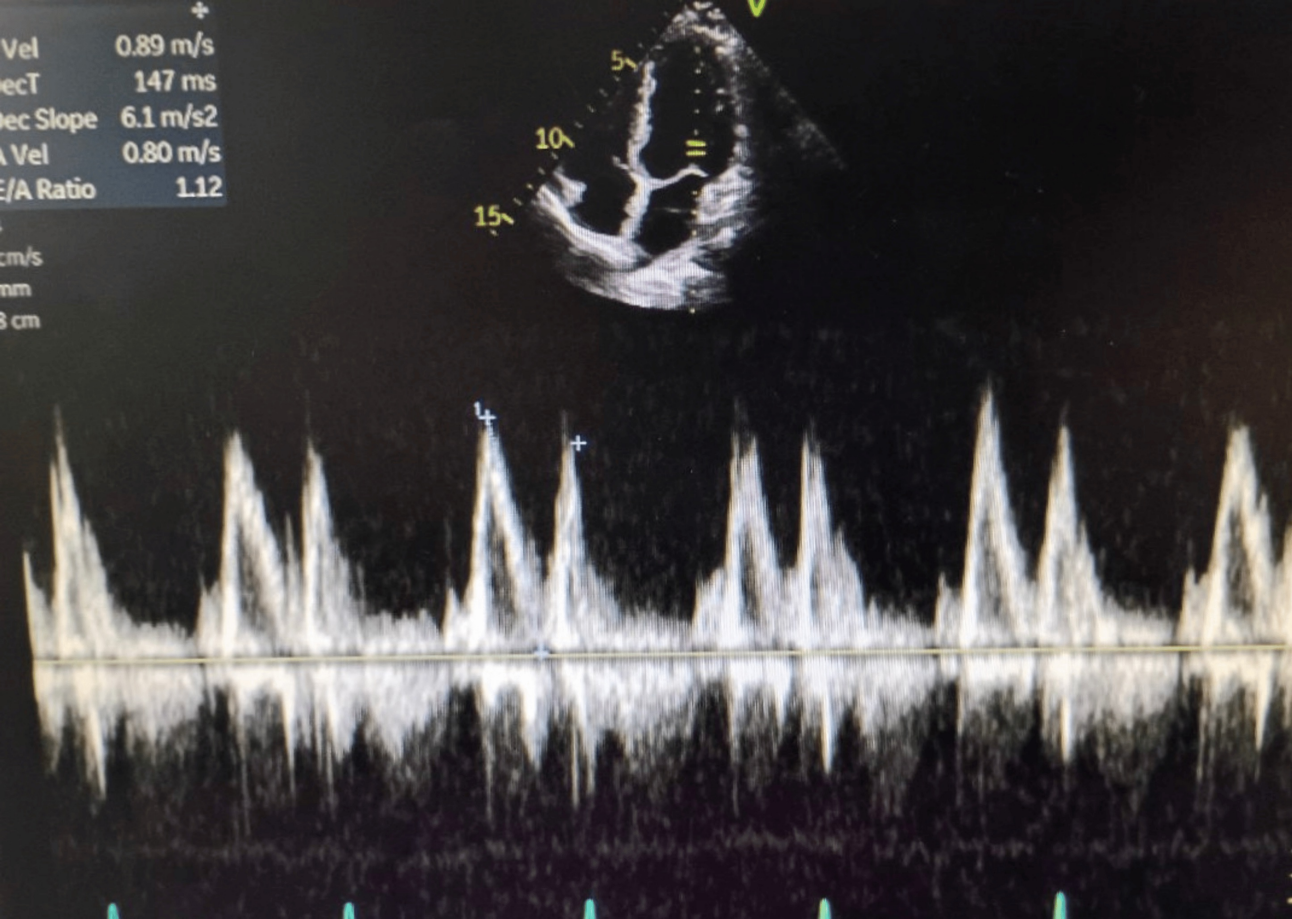
Echocardiogram showing a normal left ventricular (LV) diastolic function.

**Figure 5:**
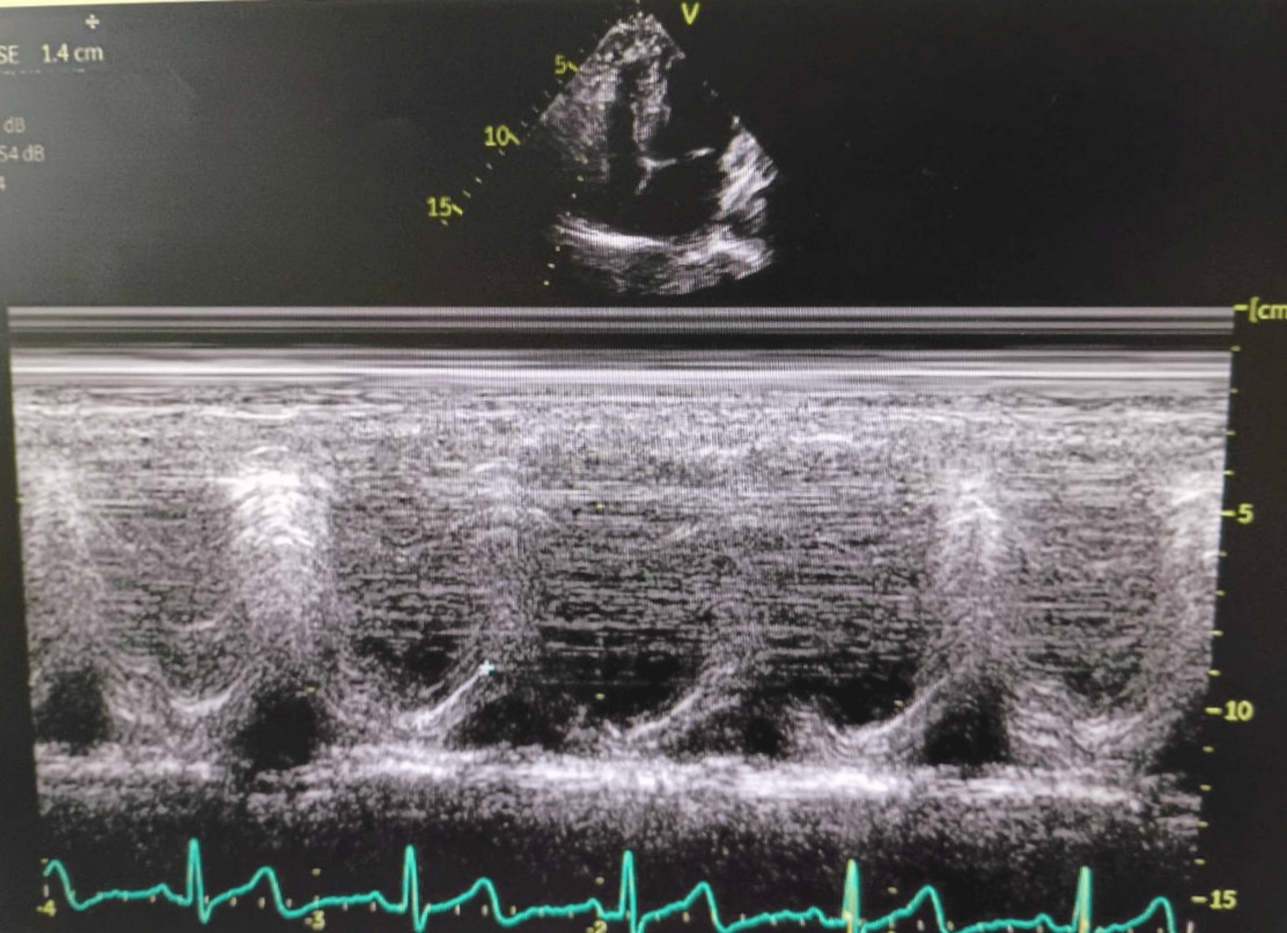
Echocardiogram showing a normal right ventricular function.

**Figure 6:**
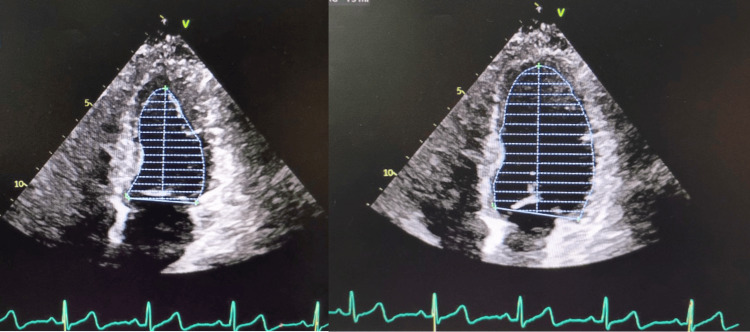
Frames of the left ventricle at the conclusion of systole & end of diastole showing a normal systolic function.

The patient reported immediate relief of symptoms. An echocardiogram was performed, demonstrating normal cardiac function across multiple parameters. Left ventricular (LF) diastolic function was within normal limits (Figure 4), as was right ventricular function (Figure 5). Additionally, LF systolic function appeared normal (Figures 6a-6b). The official echocardiogram report demonstrated normal cardiac structure and function, with no abnormalities observed in the left or right ventricles, left or right atria, mitral valve, or aortic valve. The LF ejection fraction was within normal limits, measured at 55-60%. Further blood work and lab testing were conducted. The results were thyroid panel and electrolytes that were within normal limits. It is critical to evaluate thyroid status in patients presenting with new/refractory symptoms as thyroid abnormalities can cause arrhythmias.

The Cardiology team was consulted and recommended starting a beta-blocker. The patient was scheduled for an outpatient follow-up with an electrophysiology (EP) specialist for further cardiac workup and an EP study for possible ablation. The EP study was indicated in this patient based on the patient’s history of various episodes/refractory arrhythmia. The EP study can be followed by radioablation if the source of the arrhythmia is discovered to be an accessory pathway.

## Discussion

SVT is a common arrhythmia that can present with symptoms similar to other medical conditions, such as panic attacks, making its diagnosis challenging [6]. This case report illustrates the potential for misdiagnosis of SVT as panic attacks, a frequent occurrence in clinical practice, and highlights the importance of a thorough evaluation, especially in patients with recurrent episodes of palpitations and associated symptoms.

SVT is characterized by a rapid heart rate originating above the ventricles, with the most common form being AVNRT [2]. It is often seen in young, otherwise healthy individuals, though it can occur in individuals of any age. Patients with SVT commonly experience symptoms such as palpitations, dizziness, shortness of breath, and chest discomfort, which overlap with those seen in anxiety and panic disorders. This overlap can lead to misdiagnosis and delay in appropriate treatment, as was the case with our patient. Lessmeier et al. have highlighted the significant number of patients who are initially misdiagnosed with psychiatric conditions, such as panic disorder, when they actually have underlying arrhythmias, including SVT [7]. Our case reports aim to highlight the significance of such misdiagnosis and how future steps can be implemented to decrease the number of misdiagnosed arrhythmias.

The IPED study, a multi-centre randomized controlled trial, explored diagnostic strategies for patients presenting with palpitations and pre-syncope to emergency departments. It found that the addition of a smartphone-based event recorder alongside standard care significantly improved the diagnosis of arrhythmias, including SVT, in patients with unexplained palpitations. The study highlighted the value of continuous monitoring in diagnosing arrhythmias that may be missed during a brief emergency department evaluation [2]. This finding supports the notion that patients like ours, who present with palpitations and are initially misdiagnosed with panic attacks, could benefit from advanced monitoring techniques to avoid delays in diagnosing arrhythmias.

Adding another layer to evolving diagnostic strategies, Higuchi et al. utilized convolutional neural networks (CNNs) to interpret surface electrocardiograms and identify SVT mechanisms with remarkable accuracy [8]. This AI-based approach demonstrated the potential to not only detect the presence of SVT but also distinguish between its subtypes, such as AVNRT and atrioventricular reciprocating tachycardia (AVRT), which may otherwise require invasive electrophysiological studies for differentiation. Such advancements underscore the future promise of machine learning as a clinical tool to enhance the early recognition of arrhythmias - especially when traditional ECG interpretation is inconclusive or when symptoms are nonspecific. In the case of our patient, a tool such as this would have saved the patient the multiple ER trips, the constant worrying about their diagnosis, and would have gotten them the care they needed earlier.

In this report, a 45-year-old woman with a known history of panic attacks was initially misdiagnosed with recurrent panic episodes despite presenting with palpitations, dizziness, and a sense of impending doom. The fact that her symptoms lasted for several hours and were not alleviated by anxiolytics further complicated the clinical picture. Her heart rate of 217 BPM with a regular rhythm upon presentation was a key clinical clue that ultimately led to the diagnosis of SVT. An ECG revealed narrow complex tachycardia at a rate of 225, which is consistent with AVNRT. This finding, combined with the patient's clinical history and the lack of improvement with anti-anxiety medications, made SVT the most likely diagnosis.

The initial misdiagnosis of panic attacks highlights the importance of considering a broad differential diagnosis in patients presenting with symptoms of anxiety, especially when these symptoms are unexplained or refractory to treatment. A study by Lessmeier et al. found that many patients with SVT were initially treated for anxiety disorders, resulting in a delay in appropriate management [7].

In our patient, once the correct diagnosis of SVT was made, treatment involved the administration of intravenous diltiazem, a calcium channel blocker, which successfully terminated the arrhythmia. This clinical decision was determined by the cardiology team on call. It is, however, important to note that adenosine can also be used in this case. This rapid response is consistent with the typical management of SVT, which includes vagal maneuvers, pharmacologic therapy (e.g., diltiazem or adenosine), and, in refractory cases, electrical cardioversion. In this case, vagal maneuvers were unsuccessful, but diltiazem rapidly restored normal sinus rhythm, providing immediate symptom relief. Similar cases have demonstrated the efficacy of diltiazem in the acute management of SVT [9].

Further evaluation with an echocardiogram revealed a structurally normal heart, and the patient’s thyroid function and electrolytes were within normal limits. These investigations helped rule out secondary causes of arrhythmia, further supporting the diagnosis of idiopathic AVNRT. The cardiology team recommended initiating a beta-blocker for long-term management and referred the patient for outpatient follow-up with an electrophysiologist. Given the patient’s recurrent episodes and the potential for future symptomatic arrhythmias, an EP study for possible catheter ablation can also be considered. Catheter ablation is a curative treatment for AVNRT and has shown high success rates in eliminating the arrhythmia permanently [4].

This case underscores the importance of careful clinical assessment and the need to consider cardiac arrhythmias as a potential cause of symptoms that overlap with psychiatric disorders. Early recognition of SVT and prompt initiation of appropriate treatment can significantly improve patient outcomes and reduce the need for multiple emergency department visits. Future studies examining the use of different methods, including AI-based methods, to decrease misdiagnosed arrhythmias in patients with psychiatric diagnoses may help guide clinical practices and improve diagnostic accuracy.

## Conclusions

This case underscores the importance of considering cardiac causes in patients presenting with palpitations and anxiety-like symptoms. SVT can mimic panic attacks, leading to misdiagnosis and inappropriate treatment, unnecessary exposure to medications, and prolonged patient distress. In this case, the patient's persistent symptoms despite anxiolytic therapy served as a red flag. Early recognition and appropriate management are crucial for improving patient outcomes. The recognition of narrow complex tachycardia on an ECG during symptomatic episodes was pivotal in correcting the diagnosis and initiating the appropriate treatment. Catheter ablation is a highly effective and minimally invasive procedure, resolved the patient's symptoms, and significantly improved her quality of life.

Healthcare providers should maintain a high index of suspicion for cardiac arrhythmias in patients with recurrent palpitations, especially when symptoms are refractory to psychiatric treatment. This case emphasizes the importance of comprehensive evaluations to prevent misdiagnoses and avoid unnecessary complications, thereby facilitating prompt and effective interventions. This case report also highlights one of the up-and-coming methods of integrating AI-based technology that will help decrease the number of misdiagnosed patients in the future.
